# Factors Associated with SARS-CoV-2 Infection in Fully Vaccinated Nursing Home Residents and Workers

**DOI:** 10.3390/v16020186

**Published:** 2024-01-25

**Authors:** Jesús Mateos-Nozal, Mario Rodríguez-Domínguez, Jesús San Román, Francisco Javier Candel, Noelia Villarrubia, Nuria Pérez-Panizo, Esther Segura, Juan Manuel Cuñarro, Manuel V. Mejía Ramírez-Arellano, Rafael Rodríguez-Ramos, Roberto Pariente-Rodríguez, Luisa M. Villar, Primitivo Ramos, Rafael Cantón, Alfonso J. Cruz-Jentoft, Juan Carlos Galán

**Affiliations:** 1 Servicio de Geriatría, Hospital Universitario Ramón y Cajal, IRYCIS, 28034 Madrid, Spain; nuriamaria.perez@salud.madrid.org (N.P.-P.); manuelvicente.mejia@salud.madrid.org (M.V.M.R.-A.); alfonsojose.cruz@salud.madrid.org (A.J.C.-J.); 2 Servicio de Microbiología, Hospital Universitario Ramón y Cajal, IRYCIS, 28034 Madrid, Spain; mariojose.rodriguez@salud.madrid.org (M.R.-D.); rafael.canton@salud.madrid.org (R.C.); juancarlos.galan@salud.madrid.org (J.C.G.); 3 Centro de Investigación Biomédica en Red en Epidemiología y Salud Pública (CIBERESP), Instituto de Salud Carlos III, 28029 Madrid, Spain; 4 Laboratorio Regional de Salud Pública, 28053 Madrid, Spain; jesus.sanroman@urjc.es; 5 Servicio de Microbiología Clínica y Enfermedades Infecciosas, Hospital Clínico San Carlos, 28040 Madrid, Spain; franciscojavier.candel@salud.madrid.org; 6 Servicio de Inmunología, Hospital Universitario Ramón y Cajal, IRYCIS, 28034 Madrid, Spainrafael.rodriguez.ramos@salud.madrid.org (R.R.-R.); roberto.pariente@salud.madrid.org (R.P.-R.); luisamaria.villar@salud.madrid.org (L.M.V.); 7 Residencia de Mayores Manoteras, 28050 Madrid, Spain; esther.segura@madrid.org; 8 Residencia de Mayores Adolfo Suárez, 28032 Madrid, Spain; jmcunarroal@upsa.es; 9 Agencia Madrileña de Atención Social, 28036 Madrid, Spain; primitivo.ramos@madrid.org; 10 Centro de Investigación Biomédica en Red de Enfermedades Infecciosas (CIBERINFEC), Instituto de Salud Carlos III, 28029 Madrid, Spain

**Keywords:** COVID-19, nursing homes, immunity, vaccination

## Abstract

Persons living or working in nursing homes faced a higher risk of SARS-CoV-2 infections during the pandemic, resulting in heightened morbidity and mortality among older adults despite robust vaccination efforts. This prospective study evaluated the humoral and cellular immunity in fully vaccinated residents and workers from two nursing homes in Madrid, Spain, from 2020 to 2021. Measurements of IgG levels were conducted in August 2020 (pre-vaccination) and June and September 2021 (post-vaccination), alongside assessments of neutralizing antibodies and cellular responses in September 2021 among the most vulnerable individuals. Follow-up extended until February 2022 to identify risk factors for SARS-CoV-2 infection or mortality, involving 267 residents (mean age 87.6 years, 81.3% women) and 302 workers (mean age 50.7 years, 82.1% women). Residents exhibited a significantly higher likelihood of experiencing COVID-19 before June 2021 compared with nursing staff (OR [95% CI], 7.2 [3.0 to 17.2], *p* < 0.01). Participants with a history of previous COVID-19 infection showed more significant increases in IgG levels in August 2020, June 2021 and September 2021, alongside an increased proportion of neutralizing antibodies in the most vulnerable individuals. However, IgG decay remained the same between June and September 2021 based on the previous COVID-19 status. During the *Omicron* variant wave, residents and staff showed a similar rate of SARS-CoV-2 infection. Notably, preceding clinical or immunological factors before receiving three vaccination doses did not demonstrate associations with COVID-19 infection or overall mortality in our participant cohort.

## 1. Introduction

Nursing homes have emerged as focal points for severe acute respiratory syndrome caused by SARS-CoV-2 since the onset of the pandemic. In August 2020, over half of the residents and nearly one-third of the staff had a positive serology for SARS-CoV-2 [1], contrasting with an 11.5% prevalence in the general population within the same Spanish region [2]. Mortality rates were staggering, reaching 75% among patients over 75 years old and approximately 40% in nursing homes during the first epidemic wave [3].

After this initial wave, nursing homes faced heightened infection risks, with 62 in the Community of Madrid reporting outbreaks between July and December 2020. These outbreaks were linked to facility size and resident seroprevalence [4]. Subsequently, from August 2020 to February 2021, 30 outbreaks were reported among 39 centers in Madrid, showing an incidence rate of 11%, a 55% hospitalization rate, and a 22% mortality rate [5]. Consequently, nursing homes were prioritized in the vaccination campaign starting in late 2020 [5].

Despite vaccination efforts, older adults in nursing homes remain disproportionately susceptible to infection and severe COVID-19 due to factors such as advanced age, frailty, and high care requirements. Thus, effectively managing outbreaks in these vulnerable populations remains a healthcare challenge [6].

Adding to these concerns are the evolving dynamics of the virus. While mRNA vaccines initially exhibited strong protection against symptomatic SARS-CoV-2 infection [7], this protection progressively waned with the emergence of viral variants showing higher immune escape [8]. The prevalent SARS-CoV-2 variant from February 2021, B.1.617.2 (*Delta* variant), displayed a six-fold reduced sensitivity to serum-neutralizing antibodies and an eight-fold reduced sensitivity to vaccine-induced antibodies compared with the original wild-type Wuhan-1 strain bearing D614G [9]). However, in November 2021, a new variant, B.1.1.529 (*Omicron* variant), displaced the *Delta* variant, causing a substantial surge in global cases from December 2021 to February 2022 [10], persisting across various lineages [11]. Notably, mRNA vaccine immunogenicity against the *Omicron* variant decreased significantly, with a fourfold reduction in neutralizing activity compared to the *Delta* variant [12]. The protective efficacy against infection (30%) or symptomatic infection (43%) also diminished considerably [13]. Conversely, the overall risk of hospitalization in infections caused by the *Omicron* variant is approximately halved compared to the *Delta* variant. Several factors likely contribute to this more favorable outcome, including a higher proportion of individuals with prior COVID-19 history, reduced virulence of the *Omicron* variant [14,15,16,17], and recent administration of scheduled booster doses of the SARS-CoV-2 vaccine [18,19,20]. Indeed, the risk of hospitalization by *Omicron* is reduced by 81% among patients with three vaccine doses compared to the unvaccinated [21]. Moreover, comparing two versus three vaccine doses reveals a risk reduction of 93% (88–97%) for hospitalization, 92% for severe COVID-19, and 81% for COVID-19 mortality [22].

In this study, we investigate the clinical outcomes during the *Omicron* epidemic among residents and staff in two nursing homes. We aim to assess various factors, including comorbidities, immunological parameters at different time points and other determinants that may continue to expose highly vulnerable populations, like those living in nursing homes, to severe infections in the event of significant genetic changes in SARS-CoV-2.

## 2. Methods

### 2.1. Study Design

**Figure 1:**
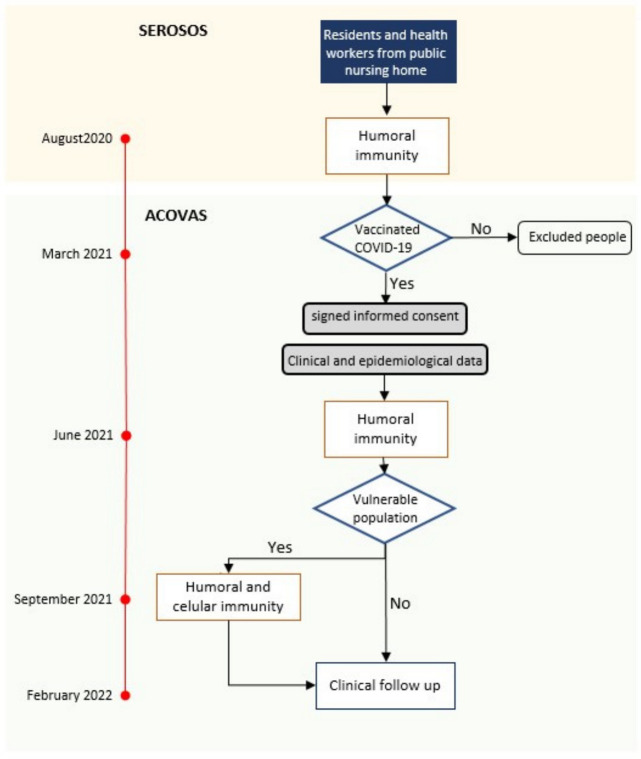
Study workflow.

This prospective study enrolled residents and staff of two nursing homes in Madrid between August 2020 and February 2022. The study involved four analysis time points, including serum sample collections at three different time points. These intervals were selected to assess immunity status before and after vaccination due to the heightened risk among these populations and the absence of specific data available at the study’s outset. Information regarding these time points is detailed in Figure 1.

First, in August 2020, as part of the SeroSOS regional study, immune globulin G against the spike protein (IgG-s levels) of the SARS-CoV-2 were measured after the first pandemic wave [1]. Second, as part of the ACOVAS study in June 2021, IgG-s levels were measured after primary vaccination. Third, in September 2021, the proportion of neutralizing antibodies against the spike protein and CD4+/CD8+ cellular immunity against SARS-CoV-2 were assessed only in the most vulnerable participants (residents or workers with IgG-s concentrations 20-fold lower than average in June 2021 or residents 85 years or older, disregarding previous antibodies levels). Finally, new cases of COVID-19 and mortality were registered between October 2021 and February 2022 (the time of the *Omicron* wave) immediately after the third dose of the vaccine.

All participants in the ACOVAS study received primary vaccination with the SARS-CoV-2 messenger (m) RNA compound (Comirnaty^®^, Pfizer-BioNTech^®^, Mainz, Germany). A first dose was administered in January or February 2021, a second dose one month later, and a booster dose in October 2021, just after the third serum sample of the study was obtained and a few weeks before the emergence of the epidemic caused by the *Omicron* variant. Ethics approval was obtained from the hospital Ethics Committee of Clinical Research (ACOVAS, Ref 127/21).

### 2.2. Population

Participants were recruited from the SeroSOS study [1] and comprised residents and staff members from two public nursing homes in Madrid who received complete vaccination and provided written informed consent. Both centers accommodated over 200 residents and various health workers, including daily physicians and nurses, and provided access to hospital medications. A new model of co-management involving workers from 31 nursing homes and a new hospital-based Geriatric Liaison Unit at Hospital Universitario Ramón y Cajal was established in 2020 [23].

Demographic and clinical data were collected from nursing home medical records and interviews with patients and staff. Two clinical outcomes during the *Omicron* epidemic were tracked: SARS-CoV-2 infection cases diagnosed by PCR Nucleic Acid Amplification test or antigen testing and mortality from nursing home records. SARS-CoV-2 infection was confirmed in participants with positive lateral flow immunochromatography (LFI) rapid antigen test or positive RNA detected by RT-PCR. Diagnostic tests were collected in the nursing homes, and nasopharyngeal swabs were sent to the reference laboratory (Hospital Universitario Ramón y Cajal) for PCR analysis. In residents, geriatric assessments, including Barthel scores for activities of daily living [24] and the Spanish-validated version of the MiniMental State exam, Lobo-MMT for cognitive function assessment [25], were performed. All participants were informed of the results of all tests, but personal data were anonymized for the analysis.

### 2.3. Laboratory Analysis

Immunological assessments were conducted at three key time points: August 2020, after the first pandemic wave (levels of IgG against the SARS-CoV-2 spike protein, IgG-s); June 2021, after two mRNA vaccine doses (IgG-s); and September 2021, before the *Omicron* epidemic (IgG-s, proportion of neutralizing antibodies against SARS-CoV-2, and CD4+/CD8+ cellular immunity against SARS-CoV-2).

Qualitative and semiquantitative (in arbitrary units per milliliter [AU/mL]) determinations of IgG against the spike (s) antigens of SARS-CoV-2 were measured by chemiluminescent microparticle immunoassays (CLIA) (Abbott^®^ Ireland Diagnostics Division, Sligo, Ireland) and the ARCHITECT System (Abbott^®^, Chicago, IL, USA). The cut-off for positivity for IgG-s was set at ≥50 AU/mL, consistent with previous validation studies [26]. For participants exceeding the upper limit of the analytical measuring interval (40,000 AU/mL), an arbitrary concentration 1.5-fold above this level was considered. The proportion of neutralizing antibodies was assessed as a surrogate marker of neutralizing activity using an immunoturbidimetric assay (SARS-CoV-2 nAb assay) based on specific antibody blockage of the interaction between the ACE2 receptor and SARS-CoV-2 RBD protein. The assay was automatized in the Alinity© System (Abbott^®^, Chicago, IL, USA) following the manufacturer’s instructions, and the cut-off value was defined as 25% inhibitory dilution (ID).

Cellular immunity was measured with the interferon-gamma (IFNγ) release assay (IGRA) using SARS-CoV-2S peptide formulations antigen1 (Ag1, lymphocytes T CD4) and antigen 2 (Ag2, lymphocytes T CD4+ and lymphocytes T CD8+) and stimulation in whole blood. The production of IFN-γ was measured using the Sandwich ELISA platform (QuantiFERON SARS-CoV-2 Research Use Only, Qiagen^®^. Hilden, Germany). The cut-off of positivity in the IGRA test used (≥15 IU/L for Ag1 and Ag2) was previously established at the Public Health Regional Laboratory of the Community of Madrid in a case–control pilot study [27].

### 2.4. Statistical Analysis

Descriptive statistics were applied to all variables, and bivariate analyses were conducted between residents and staff and across different clinical outcomes. Student’s *t*-test was used to compare normally distributed continuous variables (age, clinical scores, number of comorbidities). Mann–Whitney’s U was used for non-normally distributed variables (all laboratory parameters). The comparison of proportions for categorical variables was performed either by χ^2^ or Fisher’s exact tests. Parametric or non-parametric tests were applied as needed. The Kolmogorov–Smirnov test was used to analyze the distribution of variables. Friedman and Wilcoxon tests were used to assess the differences between repetitive measures. Multivariable logistic regression analysis used a backward model. The variance inflation factor was used to detect collinearity between variables. All statistical analyses were performed using SPSS^®^ Version 20 (IBM^®^, Chicago, IL, USA).

## 3. Results 

### 3.1. Clinical Parameters

A total of 569 participants were initially enrolled in the study, comprising 267 residents (mean [SD] age 87.6 [7.7] years old, 81.3% female) and 302 staff members (mean [SD] age 50.7 [10.3] years old, 82.1% female). The main characteristics of the study population are detailed in Table 1. As anticipated, residents exhibited a higher prevalence of comorbidities than staff members, except for immunodepression. Among residents, the median Barthel score indicated moderate dependency, and the Lobo-MMT score was indicative of moderate cognitive impairment; 187 residents (70.3%) had a diagnosis of dementia.

The likelihood of experiencing COVID-19 before June 2021 was higher among residents compared to staff members (OR [95% CI], 7.2 [3.0 to 17.2], *p* < 0.01). However, within the analysis focused on residents, no discernible clinical factors indicating a heightened risk of SARS-CoV-2 infection before either June 2021 or September 2021 were identified (Supplementary Tables S1 and S2).

### 3.2. Immunological Parameters

Table 2 outlines the various immunological parameters evaluated in our study, highlighting higher IgG-s levels and cellular immunity rates among residents compared to staff members across all assessments.

**Table 2:** Immunological parameters of nursing home residents and staff.

			**Residents (267)**	**Staff** **(302)**	***p***
August 2020	Concentration of anti-spike antibodies (mean [SD] AU/µL)		1.5 [3.2]	0.4 [1.8]	<0.01
	Proportion of positive serologies (%)		203 (76.0)	106 (35.1	<0.01
June 2021	Concentration of anti-spike IgG antibodies (mean [SD] AU/µL)		15.7 [17.9]	7.7 [12.7]	<0.01
	Positive serologies (%)		267 (100)	301 (99.7)	0.4
September 2021	Concentration of anti-spike IgG antibodies (mean [SD] AU/µL)		9.4 [12.7]	2.3 [9.4]	<0.01
	Positive serologies (%)		189 (100)	58 (100)	1
	Proportion of neutralizing antibodies (mean % [SD])		63.3 [34.1]	17.7 [17.2]	<0.01
	Celullar immunity (IGRA positivity)	CD4+ (%)	117 (62.2)	24 (39.3)	<0.01
		CD8+ (%)	71 (37.8)	7 (11.5)	<0.01
		CD4+/CD8+ (%)	123 (65.4)	24 (39.3)	<0.01

Abbreviations: SD: standard deviation, AU: arbitrary units, IGRA: interferon-gamma release assay. Additionally, the mean [SD] increase in IgG-s concentrations since 2020 was significantly higher in those with a history of COVID-19 by June 2021 (+15.3 [16.9] AU/µL vs. (+4.2 AU/µL [7.3], *p*<0.001), with consistent findings observed among residents and staff individually (Table 3).

**Table 3:** Concentration of and positivity for anti-spike IgG antibodies at different time points in nursing home residents and staff.

		History of COVID-19					
		Residents			Staff		
		Yes	No	*p*	Yes	No	*p*
No. of participants		206	59		122	164	
August 2020	Concentration of anti-spike IgG antibodies (mean [SD] AU/µL)	1.76 [3.48]	0.69 [1.30]	<0.01	0.94 [2.68]	0.07 [0.36]	<0.01
	Proportion of positive serologies (%)	83.5	52.5	<0.01	63.9	14.0	<0.01
June 2021	Concentration of anti-spike IgG antibodies (mean [SD] AU/µL)	20.65 [25.23]	8.83 [12.57]	<0.01	16.63 [22.32]	3.13 [6.81]	<0.01
	Proportion of positive serologies (%)	100	100	1	100	100	1
	Elevation in the concentration of anti-spike IgG antibodies from August 2020	18.89 [23.79]	8.14 [12.07]	<0.01	15.67 [21.21]	3.06 [6.75]	<0.01

Abbreviations: No: number, SD: standard deviation, AU: arbitrary units.

We quantified the IgG-s levels in all residents (*n* = 267) and staff members (*n* = 302) in August 2020 and June 2021). The mean [SD] IgG-s concentration in June 2021, three months post-completion of primary vaccination, was significantly higher in participants with documented prior COVID-19, including residents (20.7 [25.23] AU/µL vs. 8.8 [12.6] AU/µL, *p* < 0.001) and staff members (16.6 [22.3] AU/µL vs. 3.1 [6.8] AU/µL, *p* < 0.001).

In September 2021, IgG-s levels and IGRA tests were conducted selectively among 189 residents (70.8%) and 61 workers (29.2%) identified as more vulnerable, as previously detailed. In this cohort, IgG-s values were also higher among residents with a history of COVID-19 compared to non-infected residents (10.5 [13.7] AU/µL vs. 5.6 [7.7] AU/µL, *p* = 0.03) and in staff members with previous COVID-19 (6.0 [13.3] AU/µL vs. 1.5 [8.5] AU/µL, *p* = 0.2). Moreover, the mean [SD] temporary increase in IgG-s concentrations in this cohort was also higher among those with a history of COVID-19 (+8.3 [12.5] AU/µL vs. +3.1 [8.0], *p* < 0.001).

We also observed a higher proportion of IgG-s antibodies with neutralizing activity in September 2021 in vulnerable participants with a history of COVID-19 before June 2021 compared to those without documented infection (65.5% [33.2] vs. 27.2% [3.2], *p* < 0.01). However, the decay in IgG-s values among vulnerable participants was comparable after primary vaccination regardless of prior COVID-19 history (−45.8% [14.9] vs. −46.5% [14.4], *p* = 0.7).

Cellular immunity (CD4+ and CD8+ response against SARS-CoV-2 antigens) tested positive in 59.5% of vulnerable participants in September 2021 (123 residents and 24 staff members, *p* < 0.001). The presence of CD4+ and CD8+ responses correlated with the proportion of IgG-s antibodies exhibiting neutralizing activity (OR [95% CI], 1.02 [1.01 to 1.03], *p* < 0.01) and the documented history of COVID-19 (66.7% in participants with vs. 47.3% in participants without a recorded history of infection).

In a logistic regression analysis incorporating age, sex, personal status, and IgG-s concentration, only titers of IgG-s neutralizing antibodies remained significantly associated with cellular immunity against SARS-CoV-2 (Table 4).

### 3.3. The Omicron Epidemic

Throughout the follow-up period (October 2021 to February 2022), a total of 82 cases of COVID-19 were documented (11.9% among residents and 16.6% among workers, *p* = 0.1), with 37 recorded deaths among residents (11.6%). Higher mortality rates were observed among vulnerable residents, as detailed in Table 5.

Table 6 presents the clinical factors associated with COVID-19 incidence or mortality among residents during the follow-up period. Notably, a reduced risk of infection was observed in patients with heart disease. Conversely, higher mortality rates were evident among participants with low body weight (OR, 2.9 [95% CI, 1.4–6.1], *p* = 0.003) or active malignancy (OR, 3.6 [95% CI, 1.2–10.4], *p* = 0.01).

As indicated in Table 7, no immunological parameter exhibited a significant relationship with the risk of SARS-CoV-2 infection or mortality among residents, except for a lower CD8+ response observed in deceased participants compared to survivors (15.0% vs. 40.5% [*p* = 0.03], respectively).

## 4. Discussion

The daily incidence of COVID-19 in Madrid during the early weeks of 2022 was around 1.8% of the general population [28]. In contrast, our study found a notably higher rate of 14% among residents and workers in the two monitored nursing homes, indicating a significantly elevated risk of SARS-CoV-2 infection within these settings. Interestingly, during the *Omicron* outbreak, we observed a comparable proportion of COVID-19 cases between residents (11.9%) and staff (16.6%), a marked difference from the previous scenario, where residents had a sevenfold infection rate before June or September 2021. This shift suggests that ongoing immunization, both infections and vaccinations, might have contributed to aligning the risk of infection between older, more dependent residents and younger individuals.

Moreover, when comparing the most vulnerable participants, identified by lower IgG-s concentration in June 2021 or age exceeding 85 years, with the rest of the cohort during the *Omicron* outbreak, a similar proportion of infections emerged (13.0% versus 15.5%, respectively) (Table 7). This suggests that IgG-s titers might not serve as reliable predictors of infection risk, as suggested elsewhere [29], and that quantitative measures of cellular and immunological response might not be essential in routine clinical practice, favoring qualitative determination.

During the *Omicron* wave, the all-cause mortality rate among residents stood at 13.9%. Despite a significant number of comorbidities and moderate dependency, vulnerable residents exhibited higher overall mortality rates compared to others (10.5% versus 3.4%). This mortality rate contrasts with the 7.7% reported in Spanish nursing homes until October 2021 [30].

The decline in immunity might reduce the level of protection, particularly in older participants and for severe disease [31]. However, our cohort received a booster vaccine dose in October 2021, and a significant proportion had hybrid immunity (62.2% of residents and 43.0% of workers had experienced COVID-19), potentially averting this possibility in our cohort.

Higher IgG-s concentrations and more robust neutralizing antibody activity in vaccinated participants were linked to previous natural SARS-CoV-2 infection. Participants with higher IgG-s levels post-infection in August 2020 showed better immune responses to the vaccine in June 2021, aligning with previous findings [32]. Surprisingly, this association was absent in our most vulnerable population, contrary to previous reports [33].

Nonetheless, as others have reported [34], our cohort´s decay in IgG-s titers did not differ between vulnerable participants with or without past infection. We hypothesize that the protective effect of robust vaccination was reflected in our study by observing a similar decay in IgG-s levels across all participants. Interestingly, age did not influence the long-term antibody levels in convalescent COVID-19 patients, in contrast to previous observations [35], potentially indicating that vaccination against emergent SARS-CoV-2 variants might be the most significant prognostic during outbreaks of COVID-19. These immunological factors, added to the lower pathogenicity of the *Omicron* variant, may also explain why we could not find a single immunological or clinical factor related to the risk of SARS-CoV-2 infection or death, even in the most vulnerable group. Despite nursing homes persisting as hotspots for new SARS-CoV-2 outbreaks post-*Omicron* [36], morbidity and mortality might significantly decrease as long as the population in these facilities continues to receive booster vaccine doses, ensuring robust immunological responses [37]. The proportion of neutralizing antibodies [38] and cellular immunity [39] against SARS-CoV-2 have been proposed as protective factors against infection and its progression. However, none of these factors was associated with disease risk or mortality in our cohort. This might be attributed to the *Omicron* variant´s escape rate of 74% from neutralizing antibodies and potential evasion of humoral immunity [40].

Several limitations should be acknowledged in our study. While the cohort of residents and workers was followed from August 2020 to February 2022, complete immunological data were only available in August 2020 and June 2021. In September 2021, testing was restricted to participants considered more vulnerable. This group had 20-fold lower IgG-s concentrations, a possible risk factor for COVID-19 reinfections [41]. We opted to explore this group, as cellular immunity continues to be highly cross-reactive even after two years in the general population [42]. Moreover, we did not analyze the immune response after the booster dose because this dose was not planned during the study´s design. However, these choices potentially introduce selection bias and reduce analysis power.

Additionally, our sample size was limited to two nursing homes, each with specific characteristics, such as the number of beds, internal seroprevalence, and incidence of cases in the surrounding community [4], influencing the generalizability of findings. Clinical information during the *Omicron* outbreak was limited to case diagnoses and deaths, lacking detailed characteristics, such as severity, hospitalization, or cause of death, which would have been valuable for assessment. Furthermore, our definition of COVID-19 cases relied on antigen or PCR tests, potentially underestimating the incidence [43]. Lastly, we did not type the variants infecting participants during the study, precluding the determination of whether the infection was caused by the *Delta* or *Omicron* variants [44].

In conclusion, before the emergence of the *Omicron* variant, the risk of COVID-19 was higher among residents than nursing home staff. However, natural and post-vaccination protection, boosted by 3-dose regimens, equalized the infection risk between residents and staff. Vaccinated participants surviving the infection exhibited elevated antibody titers against SARS-CoV-2 and a higher proportion of neutralizing antibodies. However, previous infection or higher titers pre-3-dose vaccination did not correlate with new infections or overall mortality, and only anti-SARS-CoV-2 CD8 T cells were associated with mortality.

## Figures and Tables

**Table 1:** Characteristics of nursing home residents and staff.

	Residents	Staff	*p*
No	267	302	
Age (mean [SD] years)	87.6 [7.7]	50.7 [10.3]	<0.01
Females (%)	217 (81.3)	248 (82.1)	0.6
Low weight (%)	65 (24.5)	4 (1.4)	<0.01
Obesity (%)	68 (25.7)	39 (13.9)	<0.01
Hypertension (%)	180 (67.7)	55 (19.2)	<0.01
Diabetes mellitus (%)	64 (24.0)	8 (2.8)	<0.01
Dyslipidemia (%)	69 (25.9)	54 (18.9)	<0.05
COPD (%)	26 (9.8)	10 (3.5)	<0.05
Heart disease (%)	121 (45.7)	10 (3.5)	<0.01
Active malignancy (%)	18 (6.8)	2 (0.7)	<0.01
Immunodepression (%)	8 (3.0)	9 (3.1)	0.9
Barthel score (mean [SD] points)	39.0 [40.0]	--	--
Lobo-MMT score (mean [SD] points)	12.6 [10.9]	--	--
No of comorbidities (mean [SD])	3.0 [1.3]	0.7 [0.9]	<0.01
COVID-19 before June 2021 (%)	206 (77.7)	122 (42.7)	<0.01
COVID-19 before September 2021 (%)	206 (77.7)	125 (43.0)	<0.01

Abbreviations: No: number, COPD: chronic obstructive pulmonary disease, Lobo-MMT: Lobo Mini Mental test.

**Table 4:** Logistic regression analysis of factors associated with the presence of cellular immunity against SARS-CoV-2 in September 2021.

	OR (95% CI), *p*
Age (per year)	1.00 (0.98–1.03), 0.7
Sex (male vs. female)	0.53 (0.25–1.12), 0.1
History of COVID-19	1.35 (0.70–2.61), 0.4
Concentration of anti-spike antibodies (per AU/mL)	1.02 (0.99–1.06), 0.2
Proportion of neutralizing anti-spike antibodies (per point)	1.02 (1.01–1.03), <0.001

Abbreviations: AU: arbitrary units.

**Table 5:** Cases of SARS-CoV-2 infection and mortality by COVID-19 in different groups.

	All (%)	Residents (%)	Staff (%)	*p*	Vulnerable (%)	Not-Vulnerable (%)	*p*
SARS-CoV-2 infection	82 (14.4)	32 (11.9)	50 (16.6)	0.1	32 (13.0)	50 (15.5)	0.4
Any cause death	37 (6.5)	37 (13.9)	0 (0)	<0.001	26 (10.5)	11 (3.4)	0.001

**Table 6:** Clinical factors associated with SARS-CoV-2 infection or death during the Omicron wave among residents in nursing homes.

	SARS-CoV2 Infection			Any Cause Death		
	Yes	No	*p*	Yes	No	*p*
No. (%)	32 (11.9)	235 (88.1)	-	37 (13.9)	229 (86.1)	-
Females (%)	28 (87.5)	189 (80.4)	0.3	26 (70.3)	191 (83.0)	0.06
Low weight (%)	6 (18.8)	59 (25.3)	0.4	16 (44.4)	49 (21.4)	0.003
Obesity (%)	6 (18.8)	62 (26.6)	0.3	6 (16.7)	62 (27.1)	0.2
Hypertension (%)	21 (65.6)	159 (67.9)	0.8	24 (64.9)	156 (68.1)	0.7
Diabetes mellitus (%)	12 (37.5)	52 (22.1)	0.06	7 (18.9)	57 (24.8)	0.4
Dyslipidemia (%)	12 (37.5)	57 (24.4)	0.1	6 (16.2)	63 (27.5)	0.1
COPD (%)	0 (0)	26 (11.1)	0.05	2 (5.4)	24 (10.5)	0.3
Heart disease (%)	7 (21.9)	114 (48.9)	0.004	19 (52.8)	102 (44.5)	0.4
Active malignancy (%)	2 (6.3)	16 (6.9)	0.9	6 (16.7)	12 (5.2)	0.01
Immunodepression (%)	0 (0)	8 (3.4)	0.3	0 (0.0)	8 (3.5)	0.3
Barthel score (mean [SD] points)	38.9 (32.1)	39.0 (30.7)	0.9	25.6 (27.6)	41.1 (30.7)	0.1
Dementia (%)	25 (78.1)	162 (69.2)	0.3	29 (80.6)	158 (68.7)	0.1
Lobo-MMT score (mean [SD] points)	9.2 (11.2)	13.1 (10.8)	0.1	9.8 (9.8)	13.1 (11.0)	0.2
No. of comorbidities (mean [SD])	2.8 (1.2)	3.1 (1.3)	0.4	3.1 (1.3)	3.0 (1.3)	0.9
COVID-19 before June 2021 (%)	24 (75.0)	182 (78.1)	0.7	27 (75.0)	179 (78.2)	0.7
COVID-19 before September 2021 (%)	24 (75.0)	182 (78.1)	0.7	27 (75.0)	179 (78.2)	0. 7

Abbreviations: No: number, COPD: chronic obstructive pulmonary disease, Lobo-MMT: Lobo Mini Mental test.

**Table 7:** Immunological parameters associated with SARS-CoV-2 infection or death during Omicron wave among residents in nursing homes.

			SARS-CoV2 Infection			Any Cause Death		
			Yes	No	*p*	Yes	No	*p*
	No (%)		32 (12)	235 (88)	-	37 (13.9)	230 (86.1)	-
August 2020	IgG anti-spike antibodies (mean [SD] AU/µL)		1.9 [4.6]	1.4 [2.9]	0.4	1.5 [4.0]	1.5 [3.0]	0.5
	Positive serologies (%)		26 (81.3)	177 (75.3)	0.5	28 (75.7)	175 (76.1)	1
June 2021	IgG anti-spike antibodies (mean [SD] AU/µL)		13.7 [16.7]	16.0 [18.0]	0.5	14.2 [19.2]	18.5 [24.1]	0.5
	Positive serologies (%)		32 (100)	235 (100)	1	37 (100)	230 (100)	1
September 2021	IgG anti-spike antibodies (mean [SD] AU/µL)		8.4 [3.4]	9.5 [12.7]	0.7	7.1 [7.0]	10.3 [16.2]	0.5
	Positive serologies (%)		21 (100)	168 (100)	1	20 (100)	169 (100)	1
	Proportion ofneutralizing antibodies (mean % [SD])		62.4 [33.0]	63.4 [34.3]	0.9	63.7 [38.3]	63.2 [33.7]	0.9
	Cellular immunity (positivity):	CD4+ (%)	14 (66.7)	103 (61.7)	0.7	13 (65.0)	104 (61.9)	0.8
		CD8+ (%)	9 (42.9)	62 (37.1)	0.6	3 (15.0)	68 (40.5)	0.03
		CD4+/CD8+ (%)	14 (66.7)	109 (65.3)	0.9	13 (65.0)	123 (65.4)	1

Abbreviations: No: number, SD: standard deviation, AU: arbitrary units.

## Data Availability

The data that support the findings of this study are available from the corresponding author on reasonable request.

## Supplementary Materials

The following supporting information can be downloaded at: https://www.mdpi.com/article/10.3390/v16020186/s1, Table S1: Factors associated with COVID-19 before June 2021, Table S2: Factors associated with COVID-19 before September 2021.
