# Supplementary material for: Factors Associated with SARS-CoV-2 Infection in Fully Vaccinated Nursing Home Residents and Workers
Source: Viruses. 2024 Jan 25;16(2):186. doi: 10.3390/v16020186 (PMC10891794; doi:10.3390/v16020186)
Supplement: Supplementary file 1 [file viruses-16-00186-s001.zip › viruses-2839656-supplementary.pdf]

Supplementary Table S1. Factors associated with COVID-19 before June 2021

	COVID-19 before June 2021					
	Residents			Staff		
	Yes (n=206)	No (n=59)	p	Yes (n=122)	No (n=164)	p
Hypertension (%)	140 (68.0)	39 (66.1)	0.79	20 (16.4)	35 (21.3)	0.29
Diabetes mellitus (%)	46 (22.3)	17 (28.8)	0.30	2 (1.6)	6 (3.7)	0.28
Dyslipidemia (%)	50 (24.3)	19 (32.2)	0.22	25 (20.5)	29 (17.7)	0.55
COPD (%)	23 (11.2)	2 (3.4)	0.07	5 (4.1)	5 (3.0)	0.63
Heart disease (%)	92 (44.9)	29 (49.2)	0.56	4 (3.3)	6 (3.7)	0.86
Active malignancy (%)	14 (6.8)	3 (5.1)	0.63	1 (0.8)	1 (0.6)	0.83
Immunodepression (%)	6 (2.9)	2 (3.4)	0.85	4 (3.3)	5 (3.0)	0.91
Low weight (%)	46 (22.4)	19 (32.2)	0.12	2 (1.7)	2 (1.2)	0.76
Obesity (%)	57 (27.8)	11 (18.6)	0.16	21 (17.6)	18 (11.1)	0.12
Dementia (%)	141 (68.8)	45 (76.3)	0.27	--	--	--
Mean [SD] no. of comorbidities	2.99 [1.34]	3.15 [1.20]	0.4	--	--	--
Mean [SD] Barthel score	40.4 [30.7]	32.9 [29.9]	0.1	--	--	--
Mean [SD] Lobo-MMT score	13.0 [11.2]	11.1 [9.9]	0.2	--	--	--
IgG-S concentration in August 2020 (mean [SD] AU/µL)	1.76 [3.48]	0.69 [1.30]	<0.01	0.94 [2.68]	0.07 [0.36]	<0.01

Supplementary Table S2. Factors associated with COVID-19 before September 2021

	COVID-19 before September 2021					
	Residents			Staff		
	Yes (n=206)	No (n=59)	p	Yes (n=125)	No (n=166)	p
Hypertension (%)	140 (68.0)	39 (66.1)	0.79	21 (16.8)	34 (21.1)	0.36
Diabetes mellitus (%)	46 (22.3)	17 (28.8)	0.30	2 (1.6)	6 (3.7)	0.28
Dyslipidemia (%)	50 (24.3)	19 (32.2)	0.22	25 (20.0)	29 (18.0)	0.67
COPD (%)	23 (11.2)	2 (3.4)	0.07	5 (4.0)	5 (3.1)	0.68
Heart disease (%)	92 (44.9)	29 (49.2)	0.56	4 (3.2)	6 (3.7)	0.81
Active malignancy (%)	14 (6.8)	3 (5.1)	0.63	1 (0.8)	1 (0.6)	0.86
Immunodepression (%)	6 (2.9)	2 (3.4)	0.85	4 (3.2)	5 (3.1)	0.96
Low weight (%)	46 (22.4)	19 (32.2)	0.12	2 (1.6)	2 (1.3)	0.79
Obesity (%)	57 (27.8)	11 (18.6)	0.16	21 (17.2)	18 (11.3)	0.16
Dementia (%)	141 (68.8)	45 (76.3)	0.27	--	--	--
Mean [SD] no. of comorbidities	2.99 [1.34]	3.15 [1.20]	0.4	--	--	--
Mean [SD] Barthel score	40.4 [30.7]	32.9 [29.9]	0.1	--	--	--
Mean [SD] Lobo-MMT score	13.0 [11.2]	11.1 [9.9]	0.2	--	--	--
IgG-S concentration in August 2020 (mean [SD] AU/µL)	1.76 [3.48]	0.69 [1.30]	<0.01	0.92 [2.65]	0.09 [0.44]	<0.01
IgG-S concentration in June 2021 (mean [SD] AU/µL)	20.65 [25.22]	8.83 [12.57]	<0.01	16.26[22.18]	3.27 [6.81]	<0.01
